# Identification and Analysis of Graft-Responsive miRNAs in Mulberry Rootstock–Scion Interactions

**DOI:** 10.3390/genes17091024

**Published:** 2026-08-28

**Authors:** Jin Huang, Cui Yu, Wen Den, Fan Wu, Fangyuan Song, Yan Mao, Zhongcheng Zhou, Yong Li

**Affiliations:** 1 Cash Crops Research Institute, Hubei Academy of Agricultural Sciences, Wuhan 430064, China; huang_jin@hbaas.com (J.H.); yucui_b2020@163.com (C.Y.); dengwen@hbaas.com (W.D.); wufan@hbaas.com (F.W.); 18754861147@163.com (F.S.); 2 School of Ecology and Environment, Hubei Ecology Polytechnic College, Wuhan 430200, China; maoyan2156@163.com

**Keywords:** grafting, rootstock–scion interaction, mulberry, miRNA-sequencing, qRT-PCR validation

## Abstract

Grafting profoundly influences fruit tree performance, yet the molecular mechanisms underlying rootstock–scion interactions in mulberry (*Morus multicaulis*) remain poorly understood. To investigate the regulatory networks linking grafting to scion physiology, we performed an integrated analysis combining small RNA sequencing, transcriptome-based KEGG pathway assessment, and qRT-PCR validation of key target genes across five rootstock–scion combinations. High-throughput miRNA profiling of phloem tissues identified 71 conserved and 156 novel miRNAs, which exhibited distinct, genotype-dependent expression patterns. Recurrent activation of nutrient- and stress-responsive families (e.g., miR399, miR397, and miR395) was observed. Target prediction and functional enrichment analyses revealed that these miRNAs likely regulate pathways related to metabolic processes, hormone signaling, cell wall modification, and stress responses. The expression trends of their predicted target genes—including TCP4, Laccase-3, and ATP sulfurylase 1—were subsequently confirmed by qRT-PCR, revealing significant rootstock-specific regulation. Collectively, this study establishes a reference framework for elucidating the molecular mechanisms underlying scion biological processes in *Morus* species, which is of great significance for improving fruit quality, enhancing abiotic and biotic stress resistance, and boosting long-term planting productivity.

## 1. Introduction

Mulberry (*Morus* spp.) is a woody perennial fruit tree with growing economic, nutritional, and ecological importance [1,2]. Among cultivated species, *Morus multicaulis*, including the cultivar ‘Zijing’, is widely used for fruit production. Improving fruit yield and quality through optimized cultivation and rootstock selection has therefore become a major research focus [3]. Mulberry fruits are rich in anthocyanins, flavonoids, vitamins, organic acids, and polysaccharides. These compounds confer strong antioxidant capacity and documented health benefits, including hepatoprotective, hypoglycemic, and antihypertensive effects [1,4,5]. In addition, mulberry contributes to ecological sustainability. Its extensive root system and dense canopy promote soil conservation and erosion control, supporting its use in agroforestry and ecological restoration systems [6,7]. As consumer demand increases, strategies that enhance fruit quality without compromising ecological value are increasingly important.

Grafting is a central technique in perennial fruit production. It enables the combination of elite scion cultivars with rootstocks that improve water and nutrient uptake, hormone transport, stress tolerance, and fruit quality [8,9]. In grapevine, apple, citrus, and peach, rootstocks have been shown to alter fruit size, sugar and acid composition, mineral accumulation, phenolic metabolism, and abiotic stress resistance [10,11,12,13]. Mulberry shows similar rootstock-dependent variation. Distinct graft combinations display clear differences in fruit weight, soluble solids, vitamin C content, anthocyanin accumulation, and sugar-acid balance under identical growing conditions [3]. However, the molecular basis of these effects remains unclear. Grafting induces bidirectional communication between rootstock and scion through the phloem. This exchange includes metabolites, hormones, proteins, and nucleic acids that coordinate growth and development across the graft interface [14,15]. Among these signals, small RNAs have emerged as key regulators of long-distance communication. Plant microRNAs (miRNAs) and small interfering RNAs (siRNAs) can move systemically through the phloem and regulate gene expression in distant tissues [16].

Mobile miRNAs play essential roles in nutrient signaling, development, and stress responses. For example, miR399 is induced by phosphate deficiency and moves from shoots to roots to regulate phosphate homeostasis through repression of PHO2 (a phosphate overaccumulator) [17,18]. miR395 responds to sulfur limitation and controls sulfate transport and assimilation by targeting ATP sulfurylase and sulfate transporter genes [19,20]. In grafted systems of tomato, potato, and citrus, graft-induced changes in miRNA expression influence hormone signaling, vascular development, carbohydrate metabolism, and graft compatibility [15,21].

In woody perennials, the role of small RNAs is amplified by extensive and long-lived vascular systems. miRNAs are present in phloem sap and function as systemic signals over long distances [22,23]. During graft union formation, plants activate wound responses that include callus formation, cell division, and differentiation into functional vascular tissues. These processes require precise regulation of cell wall remodeling, lignification, hormone balance, and redox homeostasis. miRNAs contribute directly to these pathways. Notably, miR397 targets laccase genes involved in lignin polymerization and secondary cell wall formation, influencing xylem development and graft stability [24]. In addition, some 22-nt miRNAs trigger RDR6-dependent secondary siRNA production, amplifying regulatory signals across tissues [25,26]. Given mulberry’s strong secondary growth and vascular connectivity, small RNA-mediated signaling is likely central to rootstock–scion interactions.

Transcriptome reprogramming is another hallmark of graft establishment. Grafting activates pathways involved in protein turnover, lipid and carbohydrate metabolism, phenylpropanoid biosynthesis, DNA repair, reactive oxygen species detoxification, and hormone signaling. Transcriptome analyses in grapevine, citrus, and tomato have revealed strong induction of proteasome-, peroxisome-, and endoplasmic reticulum-related pathways during graft formation, reflecting intense cellular and metabolic reorganization [27,28,29,30]. Rootstock genotype further shapes scion transcriptomic profiles, leading to differences in growth vigor, fruit quality, nutrient status, and stress tolerance in woody fruit crops [12,31,32].

Despite the agronomic importance of grafting in mulberry, its molecular regulation remains largely unexplored, and the mechanisms by which rootstocks modulate scion fruit quality are unknown. To address these gaps, this study combined high-throughput miRNA sequencing with transcriptome-based KEGG pathway analysis across five mulberry rootstock–scion combinations. Phloem tissues were sampled 10 cm above and below the graft union. We systematically characterized conserved and novel miRNAs, analyzed their expression dynamics, and predicted their target genes. These data were integrated with transcriptome pathway analyses to identify key regulatory networks underlying rootstock–scion interactions in mulberry.

## 2. Materials and Methods

### 2.1. Plant Materials

Five mulberry rootstocks were used in this study: Sangtuo No. 2 (*Morus atropurpurea* Roxb. × *M. atropurpurea* Roxb.), Guisangyou 62 (*M. atropurpurea* Roxb. × *M. atropurpurea* Roxb.), Luza No. 1 (*M. multicaulis* Perr. × *M. multicaulis* Perr.), Zheza No. 1 (*M. multicaulis* Perr. × *M. multicaulis* Perr.), and Zheza No. 3 (*M. multicaulis* Perr. × *M. multicaulis* Perr.). All rootstocks were one-year-old seedlings with a stem diameter of approximately 0.70 cm. The seedlings were provided by the Sericulture and Tea Research Institute, Zhejiang Academy of Agricultural Sciences. Field planting was conducted on 25 March 2019. Plants were arranged in north–south rows with a spacing of 0.50 m × 2.00 m. Each rootstock was grafted with the same scion cultivar to minimize environmental and genetic variation. The scion cultivar used was Chu Shen 1 (*Morus alba* L. ‘Chushen 1 hao’), a superior triploid line developed by the Institute of Economic Crops, Hubei Academy of Agricultural Sciences. This cultivar was derived from an F1 hybrid between Zhushan 3 and Yueyou 78. Chu Shen 1 produces purple fruits with a rich flavor and began fruiting in 2020. Grafting was performed between 8 July and 11 July 2019, at the established graft union.

### 2.2. Sample Assigned and Collection

Based on the experimental design of mulberry grafting and phloem sampling, a total of ten sample groups were collected, grouped by rootstock–scion combination and sampling position relative to the graft union (Figure S1). All scions were Chu Shen 1. Phloem tissues were sampled at two positions: 10 cm above the graft union (scion side) and 10 cm below the graft union (rootstock side). The sample groups were defined as follows: Z10s and Z10X corresponded to Zheza No. 1 × Chu Shen 1, sampled 10 cm above and below the graft union, respectively. Z12s and Z12X represented Zheza No. 3 × Chu Shen 1, sampled above and below the graft union. Z2s and Z2X corresponded to Sangtuo No. 2 × Chu Shen 1. Z6s and Z6X represented Guisangyou 62 × Chu Shen 1. Z8s and Z8X corresponded to Luza No. 1 × Chu Shen 1. For each combination, the suffix ‘s’ indicates samples collected above the graft union, while ‘X’ indicates samples collected below the graft union. In addition, tissue samples were collected from three representative rootstock–scion combinations (Guisangyou 62, Zhezhe No. 3 and Luzha No. 1), with three biological replicate samples collected for each group for subsequent qRT-PCR analysis. All phloem samples were collected during the peak fruit ripening period. Samples were immediately frozen in liquid nitrogen and stored at −80 °C until RNA extraction.

### 2.3. Small RNA Library Construction and miRNA Sequencing

Total RNA was extracted from phloem tissues of all samples using RNAiso plus (D9108A, Takara, China) in accordance with the manufacturers’ instruction. The RNA molecules ranged at 18–30 nt were enriched by polyacrylamide gel electrophoresis (PAGE). High purity RNA samples (OD260/OD280 values at 1.8–2.0) were used for library construction. Libraries were prepared using the TruSeq Small RNA Sample Preparation Kit (Illumina, San Diego, CA, USA) according to the manufacturer’s instructions. Single-end sequencing with a read length of 36 bp was performed on an Illumina HiSeq 2500 platform (Illumina, San Diego, CA, USA) using standard protocols. For each mulberry rootstock–scion combination, three independent biological replicates were prepared and sequenced.

### 2.4. MiRNAs Identification and Analysis

Raw small RNA reads generated on the Illumina HiSeq platform were first quality filtered. Low-quality reads, adapter dimers, low-complexity sequences, and reads shorter than 15 nt were removed using FastQC (v0.11.9). Reads derived from rRNA, tRNA, snRNA, snoRNA, and repetitive elements were also excluded. The length distribution of the remaining clean reads were annotated in the Rfam database (Release 10.1) (https://www.sanger.ac.uk/tool/rfam/, accessed on 25 March 2025) and Genbank non-coding RNA database (http://www.ncbi.nlm.nih.gov/, accessed on 25 March 2025) to remove non-coding RNA (rRNA, tRNA, snRNA, snoRNA) and degradation fragments of mRNA using Bowtie (v1.3.1). Reads ranging from 18 to 25 nt were aligned to miRBase Release 21.0 (http://www.mirbase.org/, accessed on 25 March 2025) to identify known miRNAs. Sequences identical or highly similar to annotated plant miRNAs were defined as conserved miRNAs. Reads that did not match known miRNAs were mapped to the mulberry small RNA sequencing database (NCBI SRA BioProject PRJNA227259). Potential precursor sequences were examined for typical stem-loop structures using RNAfold (v2.4.14). Novel miRNA candidates were then predicted with Mireap (v0.2). Only candidates with a minimum folding free energy index (MFEI) greater than 0.85 were retained as putative miRNA precursors.

### 2.5. Differential Expression Analyses of miRNAs

The miRNA abundance was normalized across all libraries before differential expression analysis, following a previously described method [33]. Normalized expression levels were calculated as reads per million (RPM) using the formula: RPM = (miRNA read count/total number of clean reads) × 1,000,000. Differential expression analysis of miRNAs was performed using the DESeq2 R package (v1.34.0) with raw read counts as input. Briefly, raw count matrices of miRNA expression were first normalized by size factors to account for differences in sequencing depth across libraries. Gene-wise dispersions were estimated using a local regression model, followed by a negative binomial generalized linear model fit to test for expression differences between comparison groups. miRNAs with an adjusted *p*-value (Benjamini–Hochberg correction) < 0.05 and |log_2_ fold change| ≥ 1 were considered as significantly differentially expressed.

### 2.6. Target Prediction and Analysis of Differential miRNAs

Targets of differentially expressed miRNAs were predicted using psRNATarget by aligning miRNA sequences against the MorusDB mulberry genome database (http://morus.biodb.org, accessed on 28 March 2025). The following parameters were applied: maximum expectation value ≤ 3.0; maximum mismatches ≤ 4, no gaps allowed; central mismatch for translational inhibition restricted to positions 9–11; penalty score of 0.5 for G:U wobble pairs and 1.0 for other mismatches. To infer gene function, predicted target sequences were aligned to the Arabidopsis thaliana reference database (TAIR10). Gene Ontology (GO) terms were assigned based on corresponding annotations from TAIR. It should be noted that functional transfer based on homology to Arabidopsis thaliana (TAIR10) is useful but does not necessarily establish that the corresponding genes perform identical functions in mulberry.

### 2.7. Quantitative Real-Time PCR (qRT-PCR) Validation

To validate the expression patterns of key miRNA target genes, qRT-PCR was performed. Total RNA used for miRNA sequencing was also used for this analysis. First-strand cDNA was synthesized from 1 µg of total RNA using the PrimeScript RT reagent Kit with gDNA Eraser (Takara, Japan) following the manufacturer’s protocol. Gene-specific primers were designed using Primer Premier 5.0 software (Supplementary Table S1). The mulberry actin gene was used as an internal reference for normalization. qRT-PCR reactions were carried out in a 20 µL volume containing 10 µL of 2× TB Green Premix Ex Taq II (Takara Bio Inc., Kusatsu, Japan), 0.8 µL of each primer (10 µM), 2 µL of cDNA template, and 6.4 µL of nuclease-free water. The reactions were performed on a CFX96 Touch Real-Time PCR Detection System (Bio-Rad, Hercules, CA, USA) with the following program: initial denaturation at 95 °C for 30 s, followed by 40 cycles of 95 °C for 5 s and 60 °C for 30 s. Melt curve analysis was conducted at the end of each run to verify primer specificity. Each sample was analyzed in three technical replicates, and the entire experiment was repeated with three independent biological replicates. Relative gene expression levels were calculated using the 2^−ΔΔCt^ method. Statistical analysis was performed using one-way ANOVA followed by Tukey’s honestly significant difference (HSD) post-hoc test in GraphPad Prism 5 software. Differences were considered significant at *p* < 0.05. The results are presented as mean ± standard deviation (SD) of three biological replicates.

## 3. Results

### 3.1. The miRNA Profiling Statistic and Annotation

Across the 30 small RNA libraries, a total of 383.04 Mb of sequencing data were generated, with an average of 12.8 million reads per sample. More than 98–99% of the raw reads contained identifiable 3’ adapter sequences, confirming that the adapter ligation step was highly efficient and that the small RNA fraction was successfully captured during library preparation. Read length distributions were consistent with typical miRNA datasets. Approximately 18–40% of reads were shorter than 18 nt, while only 1–5% exceeded 30 nt. After quality filtering, 55–80% of reads were retained as clean reads. This reflects overall high sequencing quality, with modest variation among samples. Such variation likely reflects biological differences rather than technical bias. Biological replicates showed similar read length distributions and filtering rates, supporting good reproducibility of library preparation. Detailed sequencing statistics for each mulberry rootstock combination are provided in Table S2.

Annotation of clean reads revealed that rRNA-derived fragments accounted for a substantial proportion of sequences, ranging from approx. 15% to 40% across samples. In contrast, tRNA-derived reads remained low, representing about 1–4% of total reads (Table S3). Reads derived from snRNA and snoRNA were rare in all libraries. This indicates minimal contamination from nuclear RNAs and confirms effective enrichment for small regulatory RNAs. Overall, these results demonstrate high library quality, with observed differences mainly driven by biological variation.

**Figure 1:**
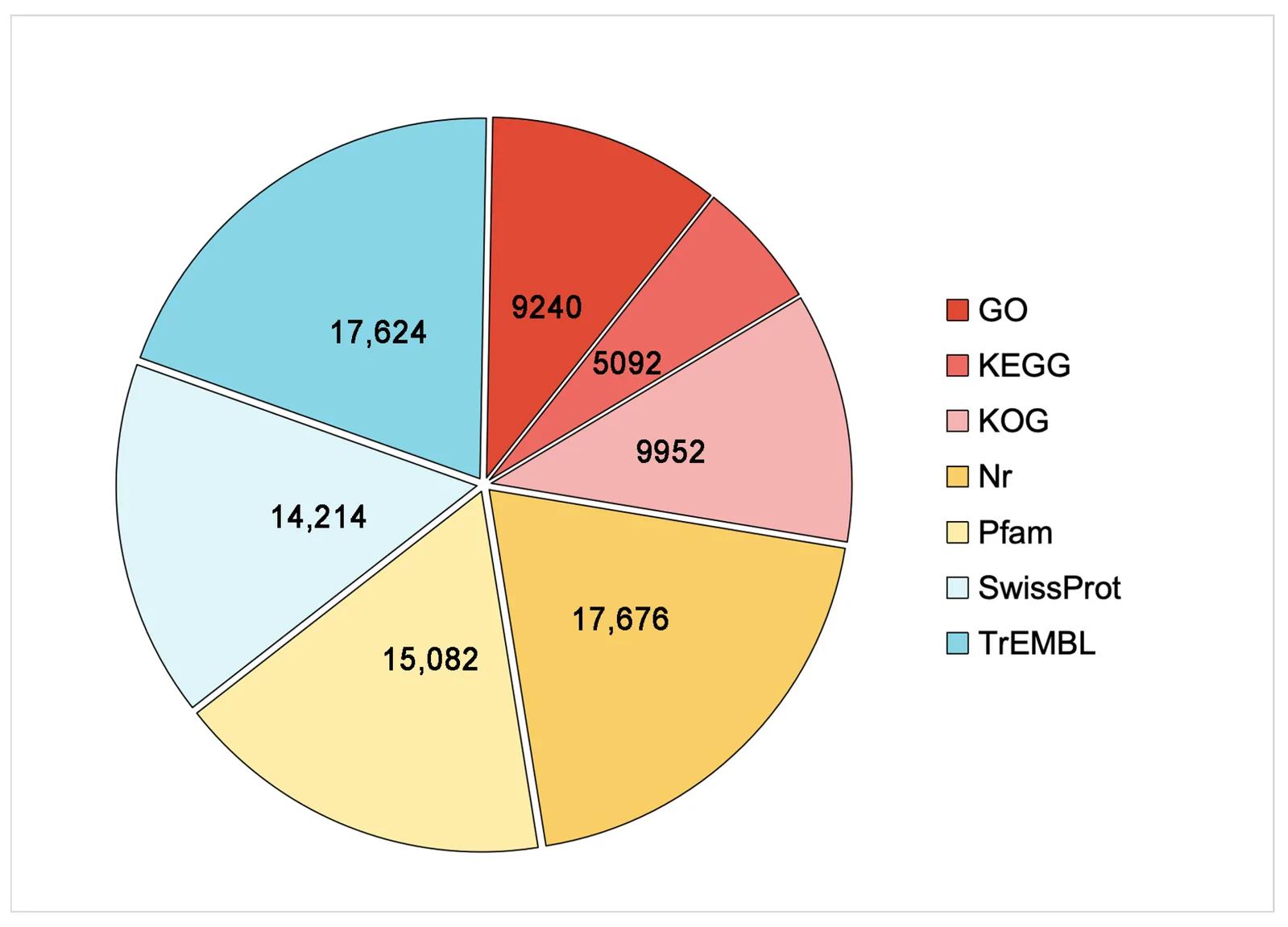
Distribution of miRNA sequencing annotations across different mulberry rootstock cultivars.

Functional annotation coverage of the miRNA sequencing data across seven reference databases is summarized in Figure 1. The largest numbers of annotated sequences were assigned to the Nr (17,676) and TrEMBL (17,624) databases. Substantial annotation was also obtained from SwissProt (14,214) and Pfam (15,082). Fewer sequences mapped to KOG (9952) and GO (9240), reflecting classification into orthologous groups and functional categories. KEGG contained the smallest number of annotated entries (5092), which is expected given the more limited scope of curated pathway databases compared with protein sequence repositories.

### 3.2. The GO, KEGG, CAZy, and Pfam Annotation and Analysis

**Figure 2:**
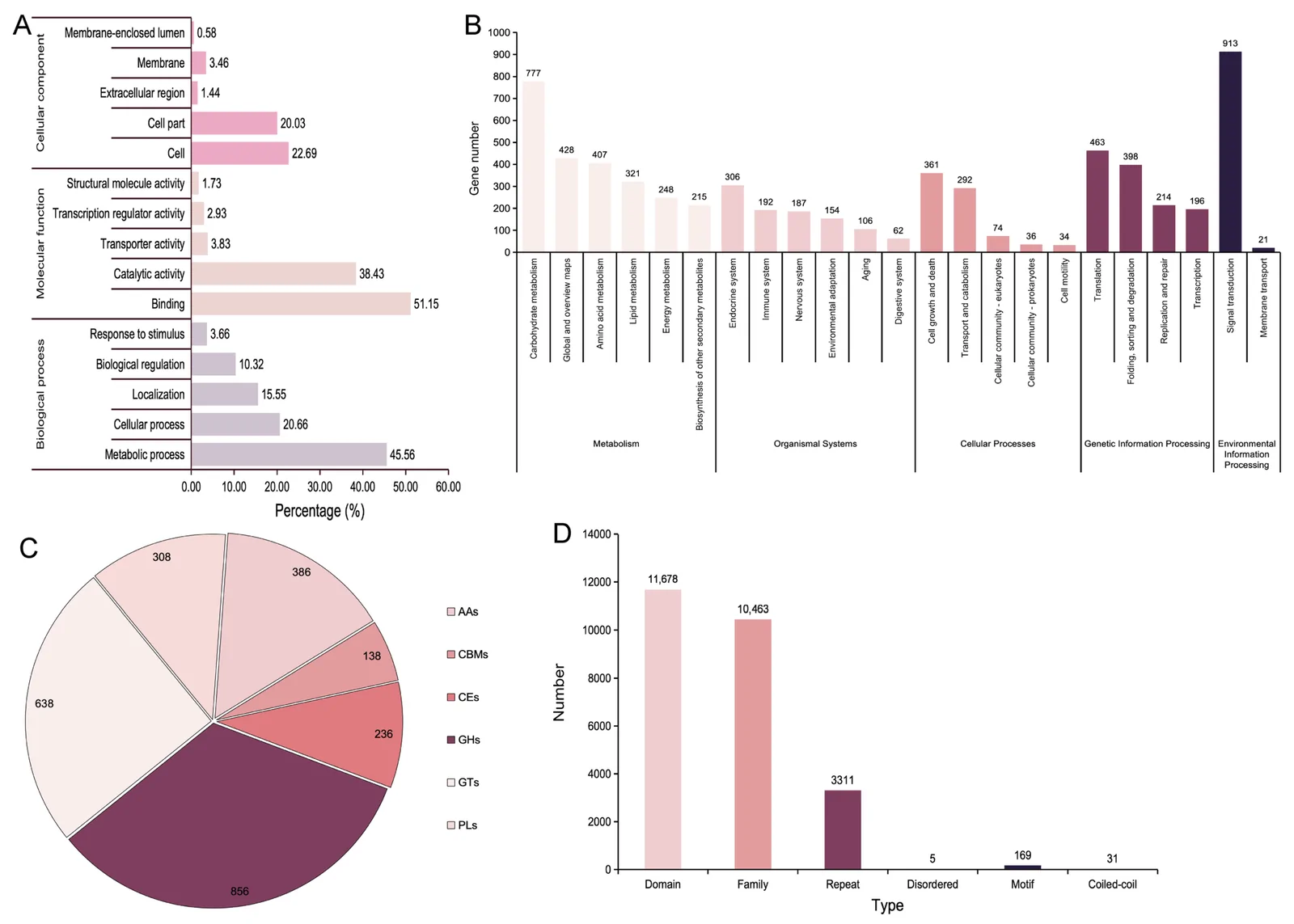
Functional annotation of miRNA sequencing data from different mulberry rootstock cultivars. (**A**) GO annotation. (**B**) KEGG pathways. (**C**) CAZy classification. (**D**) Pfam domains. Abbreviations: AAs, auxiliary activities; CBMs, carbohydrate-binding modules; CEs, carbohydrate esterases; GHs, glycoside hydrolases; GTs, glycosyltransferases; PLs, polysaccharide lyases.

GO, KEGG, CAZy, and Pfam annotations were used to characterize miRNA-associated target genes across different mulberry rootstock cultivars. In the GO annotation, genes were classified into cellular component, molecular function, and biological process categories (Figure 2A). Within the cellular component category, cell and cell part showed the highest representation, accounting for 22.69% and 20.03% of annotated genes, respectively. In the molecular function category, binding (51.15%) and catalytic activity (38.43%) were the most enriched terms. For the biological process category, metabolic process and cellular process were dominant, representing 45.56% and 20.66% of annotated genes, respectively. KEGG pathway analysis revealed that signal transduction was the most enriched pathway, with 913 annotated genes (Figure 2B). Other highly represented pathways included carbohydrate metabolism (777 genes), translation (463 genes), amino acid metabolism (407 genes), and folding, sorting, and degradation (398 genes). CAZy annotation showed that glycoside hydrolases were the most abundant enzyme class, with 856 annotated genes, followed by glycosyltransferases with 638 genes (Figure 2C). These results indicate strong representation of carbohydrate metabolism–related functions. Pfam annotation further supported this pattern. Protein domains and families accounted for the largest number of annotations, with 11,678 and 10,463 entries, respectively (Figure 2D).

### 3.3. The miRNA Identification and Analysis

**Figure 3:**
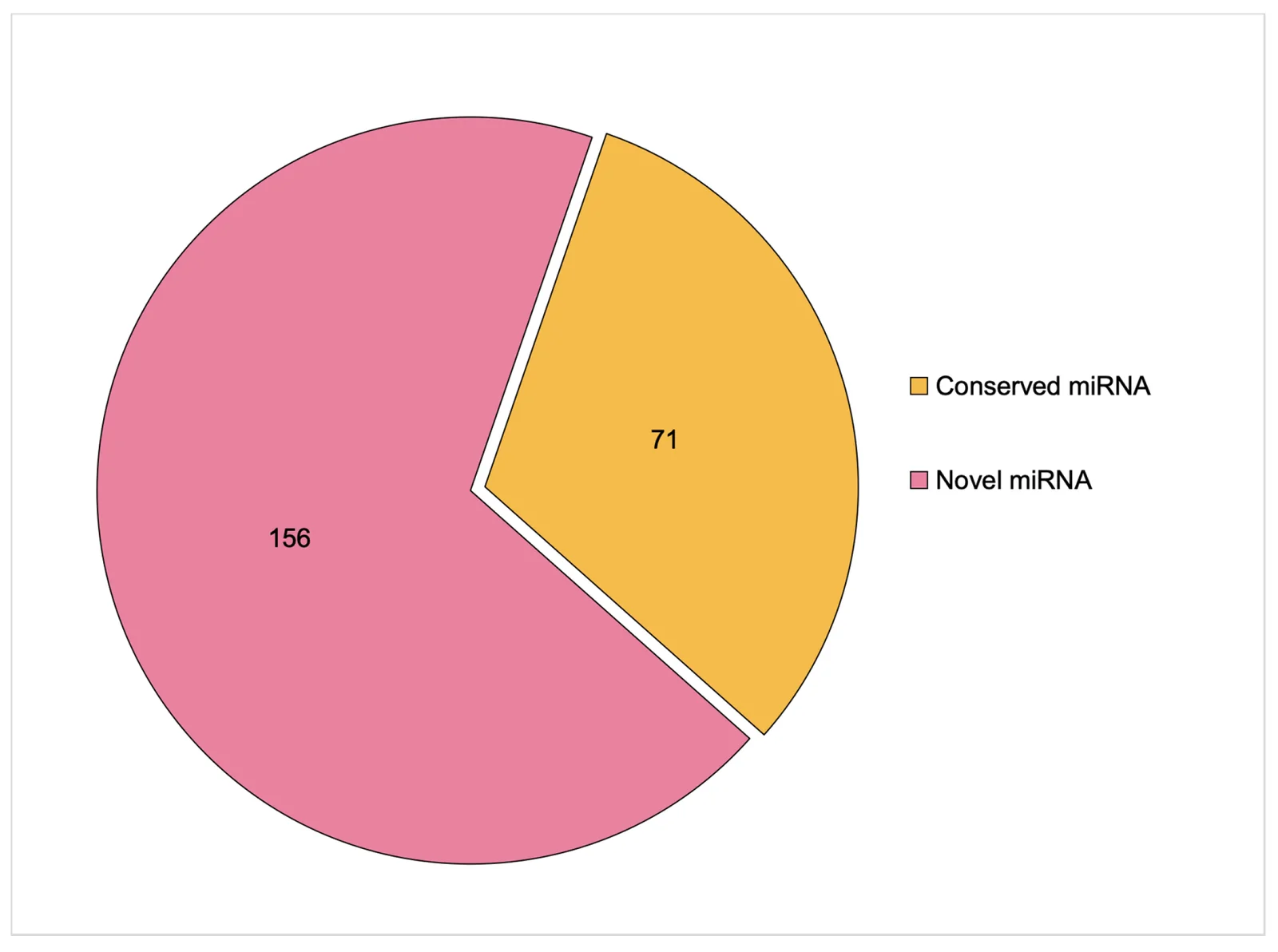
Identification of conserved and novel miRNAs across different mulberry rootstock cultivars.

**Figure 4:**
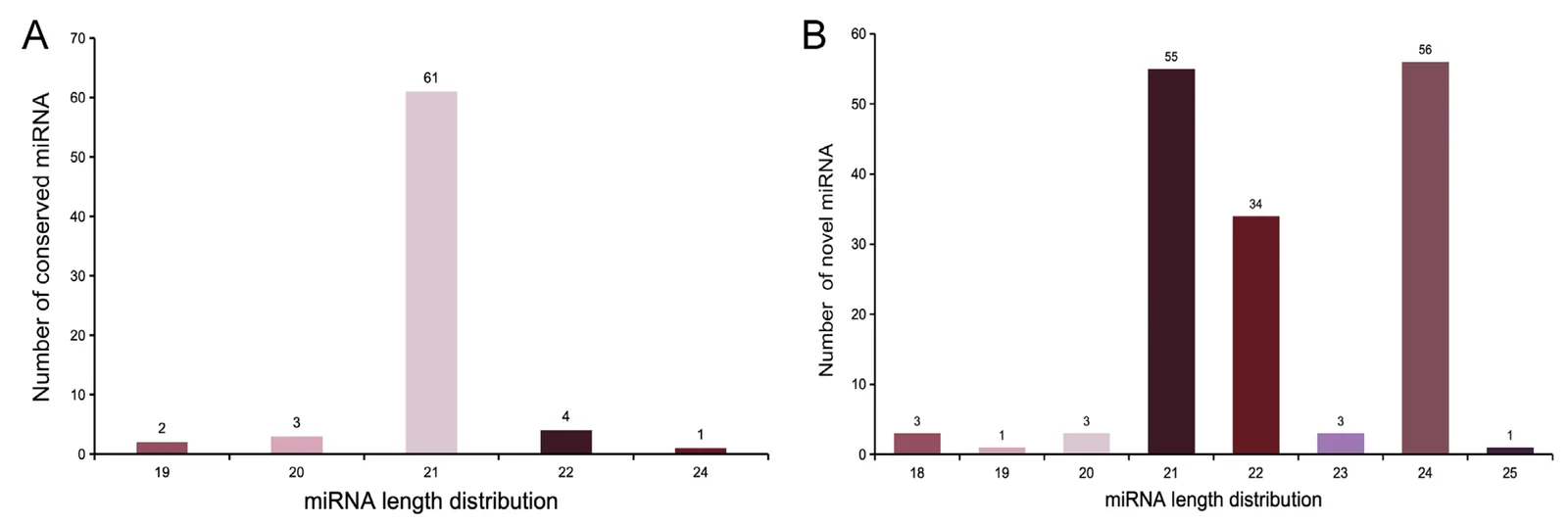
Length distribution of conserved and novel miRNAs in different mulberry rootstock cultivars. (**A**) Conserved miRNAs. (**B**) Novel miRNAs.

Further analysis identified a total of 71 conserved miRNAs and 156 novel miRNAs. The distribution of conserved and novel miRNAs across different mulberry rootstock cultivars is shown in Figure 3. Length distribution analysis revealed clear differences between conserved and novel miRNAs (Figure 4). Conserved miRNAs showed a strong enrichment at 21 nt, with 61 sequences at this length (Figure 4A). Other lengths were rare and included small numbers of 19-, 20-, 22-, and 24-nt miRNAs. This pattern is consistent with the typical size of well-characterized plant miRNAs. In contrast, novel miRNAs displayed a broader and more diverse length distribution (Figure 4B). Although 21-nt sequences remained common (55 counts), additional peaks were observed at 22 nt (34 counts) and 24 nt (56 counts). Several miRNAs were also detected at 23 nt, along with low-frequency sequences of 18, 19, 20, and 25 nt. This wider distribution suggests that many novel miRNAs may be less conserved and could represent newly evolved or lineage-specific regulatory elements. The mature sequences and predicted precursor structures of all conserved and novel miRNAs are provided in Tables S4–S8 and Figure S2.

### 3.4. The Differential Expressed miRNA Across Different Compassion Groups of Mulberry Rootstock Cultivar

**Figure 5:**
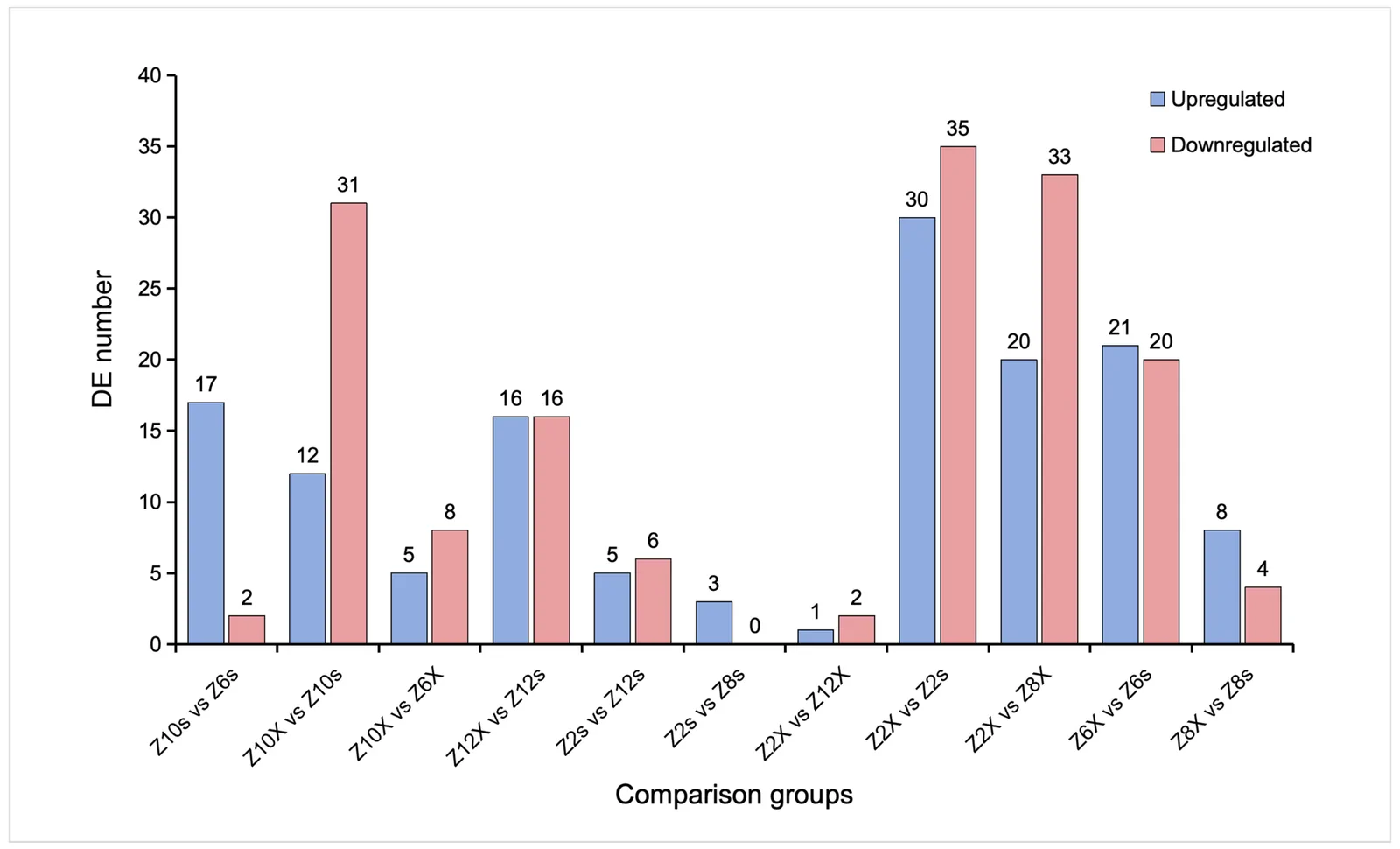
Numbers of differentially expressed miRNAs across comparison groups in different mulberry rootstock cultivars.

Figure 5 summarizes the number of differentially expressed miRNAs across multiple comparison groups and indicates whether they are upregulated or downregulated. The extent and direction of miRNA regulation varied markedly among comparisons, indicating strong context-dependent regulatory responses. Some comparisons showed moderate but clear directional changes. For example, Z10s vs. Z6s included 17 upregulated and 2 downregulated miRNAs. In contrast, Z10X vs. Z10s showed a reverse pattern, with 12 upregulated and 31 downregulated miRNAs. Z12X vs. Z12s displayed a balanced response, with 16 miRNAs upregulated and 16 downregulated. Several comparisons exhibited limited miRNA changes. These included Z2X vs. Z12X (1 upregulated, 2 downregulated), Z2s vs. Z8s (3 upregulated, none downregulated), and Z10X vs. Z6X (5 upregulated, 8 downregulated). In contrast, the largest expression shifts were observed in Z2X vs. Z2s and Z2X vs. Z8X. These comparisons showed 30 upregulated and 35 downregulated miRNAs, and 20 upregulated and 33 downregulated miRNAs, respectively. Comparisons dominated by miRNA upregulation were also observed. Z6X vs. Z6s included 21 upregulated and 20 downregulated miRNAs, while Z8X vs. Z8s showed 8 upregulated and 4 downregulated miRNAs.

### 3.5. The Analysis of DE miRNA Identified Across Different Comparison Groups

Differential expression analysis revealed distinct miRNA regulatory patterns across comparison groups, with several miRNA families repeatedly involved in rootstock- and position-dependent contrasts. These patterns indicate shared regulatory themes as well as comparison-specific responses. In the comparisons Z2s vs. Z8s and Z2s vs. Z12s, members of the miR399 family, including zma-miR399e-5p, were consistently upregulated. Predicted targets included glycerol-3-phosphate acyltransferases and TCP transcription factors, suggesting regulation of metabolic processes and transcriptional control. In Z2X vs. Z8X, several pentatricopeptide repeat (PPR) protein genes showed coordinated repression. This repression coincided with increased expression of upstream miRNAs, indicating possible miRNA-mediated regulation of organelle-associated functions. The comparison Z2X vs. Z12X showed selective induction of regulatory miRNAs, including eun-miR397a-5p. This miRNA was predicted to target laccase genes, implying reduced lignin-related activity. Consistent with this pattern, members of the miR397 family were also upregulated in Z10s vs. Z6s and were again predicted to suppress laccase isoforms. Additional regulatory shifts were observed in Z10X vs. Z6X and Z10X vs. Z10s. These transitions were characterized by increased expression of bna-miR395c or Chr2_30253-associated miRNAs. Predicted targets included nodulin genes, ATP sulfurylase, and ethylene-responsive transcription factors, suggesting changes in nutrient signaling and hormone-related pathways. Among all comparisons, Z12X vs. Z12s exhibited the strongest miRNA activation. Differentially expressed miRNAs in this group were associated with transcription factors and RNA-binding proteins, indicating broad regulatory remodeling. A summary of all differentially expressed miRNAs identified across comparison groups is provided in Table 1 and Table S9.

### 3.6. The Conserved and Novel miRNA with Target Gene Identification and Analysis

The top ten miRNAs with the highest numbers of predicted target genes were identified separately for conserved and novel miRNAs. This analysis highlights miRNAs with potentially broad regulatory roles. Among conserved miRNAs, Chr_18452 had the largest number of predicted targets, with 104 genes. This was followed by Chr2_40017 and wi_miR396b, which were associated with 35 and 32 target genes, respectively. A similar pattern was observed for novel miRNAs. Chr_18452 again showed the highest number of predicted targets, with 104 genes. Chr2_40017 and Chr13_175075 followed, with 35 and 36 target genes, respectively. Detailed statistics for conserved and novel miRNAs, including the number of predicted target genes across different mulberry rootstock cultivars, are provided in Table 2, Table 3 and Table S10.

### 3.7. The GO Enrichment Analysis

To investigate the functional roles of differentially expressed (DE) miRNAs across mulberry rootstock cultivars, Gene Ontology (GO) enrichment analyses were performed for each comparison group. Although the number of enriched terms varied among contrasts, several consistent patterns emerged across the Biological Process (BP), Molecular Function (MF), and Cellular Component (CC) categories. In the BP category, DE miRNAs from most comparisons—including Z2s vs. Z8s, Z2s vs. Z12s, Z2X vs. Z2s, Z6X vs. Z6s, Z10X vs. Z10s, and Z12X vs. Z12s were significantly enriched in metabolism-related terms. These included metabolic process, macromolecule metabolic process, and cellular process. Such enrichment indicates widespread miRNA involvement in core metabolic regulation. In some contrasts, such as Z2s vs. Z8s, additional enrichment of protein phosphorylation and phosphorus metabolic process suggested changes in kinase-dependent signaling pathways. In the MF category, catalytic activity and binding were the most consistently enriched terms across all comparisons. Many target genes were associated with transferase activity, ion binding, and oxidoreductase activity. Notably, enrichment of DNA-binding transcription factor activity in Z2s vs. Z12s highlights the role of miRNAs in modulating upstream transcriptional regulators. The CC category was dominated by membrane-related terms. Membrane, membrane part, and integral component of membrane were frequently enriched, indicating that many miRNA targets encode transporters, receptors, or membrane-associated signaling proteins. These proteins are central to nutrient uptake, signal perception, and intercellular communication. Enrichment of intracellular components, including protein complexes and, in some comparisons, the endoplasmic reticulum, further suggests miRNA involvement in protein processing, trafficking, and signal transduction. Together, these CC patterns align well with the BP and MF results and point to coordinated miRNA regulation of pathways important for root physiology and graft compatibility. A summary of GO enrichment results and the number of enriched DE miRNAs for each comparison group is provided in Table 4 and Table S11 and Figure S3.

### 3.8. The KEGG Enrichment Analysis

DEGs identified across all pairwise comparisons showed distinct patterns of KEGG pathway enrichment. Metabolic pathways were prominently enriched in several comparisons. These included biotin metabolism (ko00780), glycerolipid metabolism (ko00561), fatty acid biosynthesis (ko00061), methane metabolism (ko00680), and peroxisome (ko04146). Comparisons such as Z8X vs. Z8s and Z12X vs. Z12s showed especially strong enrichment of lipid-related metabolic pathways. Pathways related to genome stability and cell cycle control were also enriched in multiple stage-dependent comparisons. These included DNA replication (ko03030), mismatch repair (ko03430), homologous recombination (ko03440), and the Fanconi anemia pathway (ko03460). In later-stage contrasts, such as Z10X vs. Z10s and Z10X vs. Z6X, additional enrichment was observed for protein processing in the endoplasmic reticulum (ko04141) and estrogen signaling (ko04915). A summary of KEGG enrichment results and the numbers of enriched DEGs for each comparison group is provided in Table 5 and Table S12.

### 3.9. Validation of Key Target Gene Expression by qRT-PCR

To experimentally verify the regulatory effects of graft-responsive miRNAs on their predicted targets, we selected five key candidate genes for qRT-PCR analysis across three representative rootstock–scion combinations: Guisangyou 62, Zheza No. 3, and Luza No. 1. The genes included TCP4 (a transcription factor), *Laccase-3* (LAC3, involved in lignin biosynthesis), *ATP sulfurylase 1* (ATPS1, central to sulfur metabolism), LEP (an ethylene-responsive transcription factor), and *Glycerol-3-phosphate acyltransferase 6* (GAPT6, implicated in lipid metabolism) [34,35,36,37].

**Figure 6:**
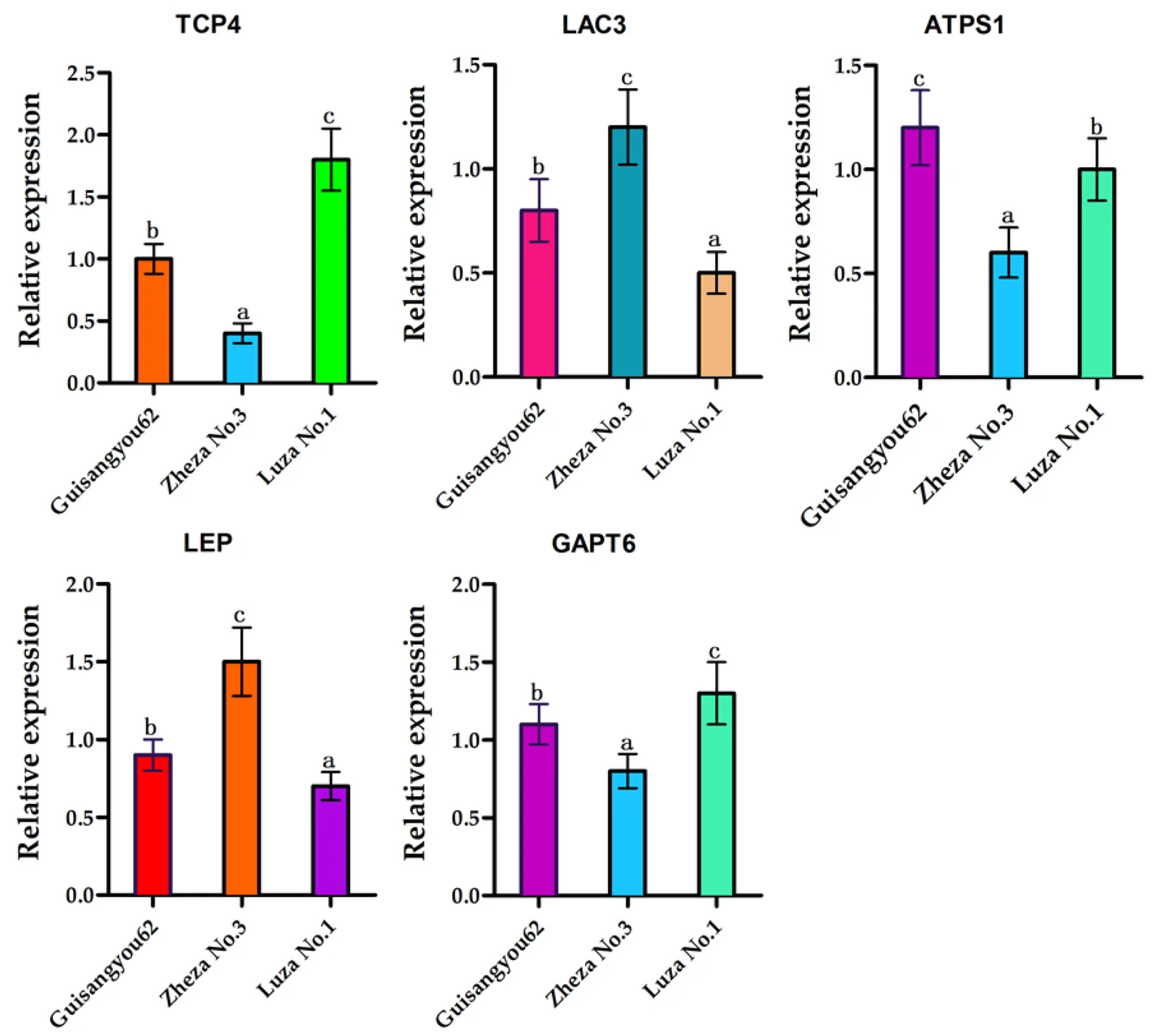
Validation of key target gene expression by qRT-PCR in three representative rootstock–scion combinations. Data represent means ± SD of 3 repeats. Different letters above the bars indicate significant differences, as determined using a Tukey honest significant difference test (*p* < 0.05).

The expression patterns revealed distinct rootstock-dependent regulation (Figure 6). Notably, TCP4 expression was highest in the Luza No. 1 combination (1.8-fold relative to Guisangyou 62), which aligns with its previously reported role in development and may correlate with the higher titratable acidity observed in fruits from this rootstock. Conversely, LAC3 showed peak expression in the Zheza No. 3 combination (1.5-fold), potentially contributing to enhanced graft union lignification and stability. ATPS1 was most highly expressed in Guisangyou 62, consistent with its role in sulfur assimilation, which may influence flavor compound synthesis. The ethylene-responsive factor LEP was also upregulated in Zheza No. 3 (1.5-fold), possibly linking ethylene signaling to the high soluble solid content characteristic of this graft combination. Expression of GPAT6 was elevated in Luza No. 1, suggesting modifications in membrane lipid metabolism that could impact stress adaptation.

These qRT-PCR results robustly corroborate the bioinformatic predictions derived from miRNA sequencing. The confirmed inverse expression trends between specific miRNAs (e.g., miR399 and TCP4; miR397 and LAC3) and their targets exhibit expression patterns consistent with the predicted miRNA-target regulatory relationship (Table S13 and Figure S4).

## 4. Discussion

This study provides a comprehensive view of graft-responsive regulatory networks in mulberry by integrating small RNA sequencing, transcriptome-based KEGG pathway analysis, and experimental validation of key target genes via qRT-PCR across multiple rootstock–scion combinations. By combining miRNA, mRNA, and targeted expression data, we reveal a multilayered regulatory system shaped by rootstock genotype, sampling position, and developmental stage. These findings extend current knowledge of mulberry graft biology, which has largely focused on morphological and physiological traits, by uncovering and experimentally verifying molecular mechanisms that underlie rootstock–scion interactions. Our results show that miRNAs, their target genes, transcriptional pathways, and cellular processes act in a coordinated manner to regulate nutrient signaling, metabolism, stress responses, vascular reconnection, and long-term scion performance.

Small RNA sequencing generated high-quality datasets that captured the diversity of phloem small RNAs in mulberry. Variation among samples was mainly biological rather than technical. The moderate proportion of rRNA-derived reads, together with very low levels of snRNA and snoRNA contamination, is consistent with the characteristics of phloem RNA. Phloem sap often contains partially degraded ribosomal RNA due to the enucleate nature of sieve elements and the instability of vascular RNAs [38,39]. Similar small RNA profiles have been reported in woody perennials such as poplar, citrus, grapevine, and apple, especially in grafted or stress-responsive tissues [23,27,40,41]. Importantly, previous studies indicate that such variability reflects real biological responses rather than sequencing artifacts [42,43]. Together, these observations confirm that our datasets reliably represent the in vivo phloem small RNA landscape in grafted mulberry.

The identification of 71 conserved and 156 novel miRNAs highlights both evolutionary conservation and species-specific innovation within the mulberry small RNA repertoire. Conserved miRNAs were dominated by 21-nt sequences, consistent with canonical DICER-LIKE1-dependent processing observed in many dicots [44,45]. In contrast, novel miRNAs showed broader length distributions, with substantial numbers of 22-nt and 24-nt species. Such patterns are typical of recently evolved MIR loci with more flexible processing features, as reported in soybean, Medicago, and Populus [46,47]. A considerable proportion of these 22-nt and 24-nt novel miRNAs were detected in mulberry phloem, particularly in tissues below the graft union. The enrichment of these non-canonical-length novel miRNAs at the graft interface implies that they may participate in molecular responses triggered by grafting. Together, these results indicate that mulberry harbors a dynamic small RNA system that responds to grafting stress at the phloem tissue level.

Target prediction analysis revealed that many mulberry miRNAs regulate large gene networks, consistent with their role as broad post-transcriptional regulators. Our qRT-PCR validation of five key predicted targets (Figure 6) provided significant functional support for these bioinformatic insights. miRNAs such as those targeting TCP4 (putatively via miR399), LAC3 (via miR397), and ATPS1 (via miR395) showed expression patterns that were not only rootstock-dependent but also logically inverse to the expression trends of their regulating miRNAs in specific combinations. This pattern aligns with the established function of plant miRNAs in controlling gene families rather than single targets [34]. miR396, for example, targets multiple growth-regulating factor genes and coordinates cell proliferation and organ growth in Arabidopsis and grapevine [35,36]. miR399 serves as a central regulator of phosphate homeostasis, acting systemically through repression of PHO2 and downstream transport pathways [37]. The validated downregulation of its target TCP4 in certain contexts supports its role in modulating developmental processes in grafted mulberry. Similar miRNA-centered regulatory architectures have been described in grafted citrus and cucumber, where mobile miRNAs coordinate nutrient transport, stress responses, and rootstock--scion signaling [29,48,49]. These parallels suggest that mulberry graft performance is governed by coordinated regulation of complex gene networks rather than isolated pathways.

Differential expression analysis revealed strong genotype-dependent and position-specific miRNA regulation. Comparisons such as Z2X vs. Z2s and Z2X vs. Z8X showed pronounced miRNA expression shifts, which are consistent with active signaling across the graft junction or localized responses to rootstock-derived cues. Similar asymmetric miRNA expression and long-distance movement have been reported in Arabidopsis grafting systems, where nutrient-responsive miRNAs such as miR399 and miR395 move through the phloem from source to sink tissues [23,50]. Genotype-dependent control of small RNA mobility has also been documented in grapevine heterografts [51]. The patterns observed in mulberry are consistent with these systems, suggesting a conserved role for miRNA mobility and localized biosynthesis in rootstock–scion communication across woody perennials. Several miRNA families were repeatedly differentially expressed across comparisons, suggesting conserved roles in graft biology. miR399 was consistently upregulated and is well known as a phloem-mobile signal regulating phosphate homeostasis [17]. In mulberry, the correlated suppression of its predicted target TCP4 (Figure 6) links phosphate signaling to developmental regulation, potentially influencing scion vigor. miR397 was also frequently induced, particularly below the graft union, where it likely suppresses laccase genes involved in lignin polymerization. The significant upregulation of LAC3 in the Zheza No. 3 combination, where miR397 may be less active, provides direct evidence for this regulatory axis and its potential role in enhancing graft union strength [24,52]. miR395 showed increased expression in later-stage comparisons and is known to regulate sulfur assimilation and redox balance while moving systemically through the phloem [22]. Its putative target, ATPS1, displayed a complementary expression pattern in Guisangyou 62, underscoring the role of this miRNA in fine-tuning sulfur metabolism. Together, these recurring patterns highlight conserved miRNA-mediated integration of nutrient sensing, vascular development, and stress adaptation during graft establishment. Together, these recurring patterns are consistent with a conserved role for miRNA-mediated integration of nutrient sensing, vascular development, and stress adaptation during graft establishment.

Functional enrichment analysis of miRNA targets revealed strong bias toward metabolic processes, catalytic activity, binding functions, membrane components, and intracellular signaling. These categories include enzymes, transporters, and receptors essential for nutrient allocation and hormone perception. Our qRT-PCR results validate that genes central to these categories are indeed differentially regulated. For instance, GPAT6 (lipid metabolism) and ATPS1 (catalytic activity) showed clear rootstock-specific expression. Similar enrichment patterns have been reported in grafted grapevine, citrus, pecan, and hickory, where miRNAs regulate metabolism, membrane transport, and signaling during vascular reconnection [51,53,54]. The consistent enrichment of membrane-related terms in mulberry suggests a conserved role for miRNAs in controlling membrane transport, receptor-mediated signaling, and vesicle trafficking during graft union formation.

Metabolic pathway enrichment, including lipid metabolism and peroxisome-related processes, highlights strong rootstock effects on scion physiology. The validated expression of GPAT6, a key enzyme in lipid metabolism, directly connects these pathway analyses to a functional molecular component. Peroxisomes play key roles in fatty acid β-oxidation, reactive oxygen species detoxification, and jasmonic acid biosynthesis, all of which are critical during wound repair and graft union formation [55,56]. Activation of peroxisome-associated genes has been reported during grafting in peach, apple, and poplar, supporting a conserved role for redox control and hormone balance [57,58]. Enrichment of lipid metabolism pathways also aligns with the extensive membrane remodeling required for phloem differentiation and reconnection. Studies in grapevine show that graft combinations with higher vigor display elevated lipid biosynthesis and membrane-associated gene expression [51,59].

Finally, the integration of qRT-PCR-validated gene expression data provides a robust molecular explanation for previously reported rootstock effects on mulberry fruit quality. Physiological studies have shown that rootstocks influence sugar accumulation, soluble solids, and organic acid content in mulberry fruits [60,61]. Our validation indicates that key regulators of these processes are differentially expressed. For example, the high expression of the ethylene-responsive transcription factor LEP in the Zheza No. 3 combination (Figure 6) coincides with its highest soluble solid content, suggesting a direct role for ethylene signaling in promoting sugar accumulation or ripening. Conversely, the distinct expression profile of TCP4 in Luza No. 1 may underlie its characteristic fruit acidity. Similar rootstock-driven effects on carbohydrate and acid metabolism have been widely documented in peach, citrus, and watermelon, where altered sugar transport and carbon allocation affect fruit sweetness and yield [62,63,64]. The transcriptomic shifts and their experimental validation presented here provide a robust preliminary mechanistic framework linking graft-induced metabolic reprogramming to fruit quality outcomes in mulberry.

## 5. Conclusions

Overall, this study suggests that the interaction between mulberry rootstocks and scions may be regulated by a highly coordinated miRNA–mRNA regulatory network. The functional implications of these regulatory networks have been further validated through targeted gene expression analysis. These networks collectively participate in the regulation of nutrient allocation, redox homeostasis, membrane remodeling, protein turnover, and genomic stability. These physiological processes are crucial for scion callus formation, revascularization of the vascular system, and the sustained healthy growth of the scion. Through the identification of both conserved and lineage-specific miRNAs, combined with the validation of their potential target genes, this study demonstrates that mulberry integrates ancient regulatory mechanisms with newly evolved small RNA signaling pathways during the scion response process. In conclusion, these findings provide a reference framework for understanding the molecular mechanisms underlying scion biology in mulberry species; they also offer a theoretical basis for the rational selection of rootstocks based on molecular markers, thereby improving fruit quality, enhancing stress resistance, and increasing long-term productivity.

## Figures and Tables

**Table 1:** List of differentially expressed miRNAs identified in all comparison groups.

Comparison Group	miRNA ID	Genes	log_2_ Fold Change
Z2s vs. Z8s	zma-miR399e-5p	*Transcription factor TCP4*	+4.485
	zma-miR399e-5p	*Glycerol-3-phosphate 2-O-acyltransferase 6*	+4.485
Z2s vs. Z12s	zma-miR399e-5p	*Transcription factor TCP4*	+4.398
	zma-miR399e-5p	*Glycerol-3-phosphate 2-O-acyltransferase 6*	+4.398
Z2X vs. Z2s	ptc-miR172b-5p	*Pentatricopeptide repeat-containing protein At3g46790*	+4.580
	ptc-miR172b-5p	*Annexin D5*	+4.580
	zma-miR399e-5p	*Transcription factor TCP4*	−4.821
	zma-miR399e-5p	*Glycerol-3-phosphate 2-O-acyltransferase 6*	−4.821
Z2X vs. Z8X	Chr2_36236	*WEB family protein At2g40480*	+3.189
	Chr2_36236	*Putative callose synthase 8*	+3.189
	Chr4_66775	*Pentatricopeptide repeat-containing protein At3g08820*	−4.738
	Chr4_66775	*Pentatricopeptide repeat-containing protein At4g01570*	−4.738
Z2X vs. Z12X	Chr3_54055	*Transcription factor GTE3*	+2.439
	Chr3_54055	*L-type lectin-domain containing receptor kinase IX.1*	+2.439
	eun-miR397a-5p	*Laccase-11*	−2.318
	eun-miR397a-5p	*Laccase-3*	−2.318
Z6X vs. Z6s	Chr2_30253	*Topless-related protein 3*	+4.239
	Chr2_30253	*Ethylene-responsive transcription factor LEP*	+4.239
	Chr10_141424	*Calcium-transporting ATPase 9, plasma membrane-type isoform X1*	−5.336
	Chr10_141424	*Metal tolerance protein 11*	−5.336
Z8X vs. Z8s	Chr4_66775	*Pentatricopeptide repeat-containing protein At3g08820*	+3.056
	Chr4_66775	*Transmembrane protein 53*	+3.056
	eun-miR397a-5p	*Laccase-3*	−3.614
	eun-miR397a-5p	*Laccase-17*	−3.614
Z10s vs. Z6s	eun-miR397a-5p	*Laccase-3*	+3.840
	eun-miR397a-5p	*Laccase-4*	+3.840
	vvi-miR171b	*WPP domain-interacting tail-anchored protein 1*	−1.249
	Chr10_147783	*Protein IQ-DOMAIN 14*	−1.121
Z10X vs. Z6X	bna-miR395c	*Early nodulin-93-like*	+1.486
	bna-miR395c	*ATP sulfurylase 1*	+1.486
	Chr4_66775	*Myb family transcription factor APL*	−3.512
	Chr4_66775	*Pentatricopeptide repeat-containing protein At4g01570*	−3.512
Z10X vs. Z10s	Chr2_30253	*Ethylene-responsive transcription factor LEP*	+3.274
	Chr2_30253	*Protein MICRORCHIDIA 6 isoform X1*	+3.274
	mdm-miR827	*SPX domain-containing membrane protein At4g22990*	−5.038
	mdm-miR827	*Pentatricopeptide repeat-containing protein At5g08310*	−5.038
Z12X vs. Z12s	Chr2_30253	*Topless-related protein 3*	+6.939
	Chr2_30253	*Ethylene-responsive transcription factor LEP*	+6.939
	Chr14_186856	*Pumilio homolog 4*	−3.321
	Chr14_186856	*Protein downstream neighbor of son homolog*	−3.321

**Table 2:** Summary of identified miRNAs and their predicted target genes in different mulberry rootstock cultivars.

The miRNA ID	Number of Target Genes
Chr1_18452	104
Chr2_40017	35
wi-miR396b	32
Chr13_175075	26
Chr1_5246	25
Chr2_30253	20
Chr4_66775	20
Cas-miR159a	17
Chr12_172016	16
Gma-miR319p	15

**Table 3:** Summary of novel miRNAs and their predicted target genes in different mulberry rootstock cultivars.

The miRNA ID	Number of Target Genes
Chr1_18452	104
Chr2_40017	35
Chr13_175075	26
Chr1_5246	25
Chr2_30253	20
Chr4_66775	20
Chr12_172016	16
Chr11_156754	11
Chr11_156986	11
Chr1_11852	11

**Table 4:** GO enrichment statistics and numbers of DE miRNAs for each comparison group in mulberry rootstock cultivars.

Comparison Groups	Biological Process	Molecular Function	Cellular Component
Z2s vs. Z8s	protein phosphorylation (2)	molecular_function (5)	
	phosphorylation (2)	catalytic activity (4)	
	phosphorus metabolic process (2)	transferase activity (3)	
Z2s vs. Z12s	biological_process (4)	molecular_function (6)	cellular_component (1)
	metabolic process (3)	binding (3)	membrane (1)
	cellular process (2)	DNA binding transcription factor activity (2)	membrane part (1)
Z2X vs. Z2s	biological_process (41)	molecular_function (58)	cellular_component (14)
	metabolic process (29)	binding (35)	membrane (8)
	cellular process (28)	organic cyclic compound binding (16)	intracellular part (7)
Z2X vs. Z8X	biological_process (41)	molecular_function (57)	cellular_component (15)
	metabolic process (28)	binding (38)	membrane (10)
	cellular process (26)	catalytic activity (23)	membrane part (7)
Z2X vs. Z12X	metabolic process (11)	protein complex (1)	molecular_function (13)
	biological_process (11)	macromolecular complex (1)	catalytic activity (8)
	cellular macromolecule metabolic process (6)	intracellular part (1)	binding (8)
Z6X vs. Z6s	biological_process (42)	molecular_function (58)	cellular_component (13)
	metabolic process (33)	binding (40)	membrane (8)
	cellular process (26)	catalytic activity (23)	intracellular part (7)
Z8X vs. Z8s	biological_process (18)	molecular_function (29)	cellular_component (3)
	metabolic process (16)	binding (21)	membrane (2)
	cellular process (12)	ion binding (10)	protein complex (1)
Z10s vs. Z6s	biological_process (18)	molecular_function (26)	cellular_component (4)
	metabolic process (15)	binding (15)	integral component of membrane (2)
	cellular process (10)	catalytic activity (14)	intrinsic component of membrane (2)
Z10X vs. Z6X	biological_process (19)	molecular_function (30)	cellular_component (4)
	metabolic process (14)	binding (21)	membrane (3)
	cellular process (13)	catalytic activity (15)	endoplasmic reticulum (1)
Z10X vs. Z10s	biological_process (38)	molecular_function (52)	cellular_component (12)
	metabolic process (29)	binding (33)	membrane (8)
	cellular process (20)	catalytic activity (21)	membrane part (5)
Z12X vs. Z12s	biological_process (24)	molecular_function (38)	cellular_component (7)
	metabolic process (17)	binding (25)	membrane (5)
	cellular process (15)	organic cyclic compound binding (13)	organelle (3)

Indicator: (Number) indicates the number of enriched DE miRNA.

**Table 5:** KEGG enrichment statistics and numbers of DEGs for each comparison group in mulberry rootstock cultivars.

Comparison Groups	Pathways	DEG Number
Z2s vs. Z8s	Peroxisome	1
Z2s vs. Z12s	Antigen processing and presentation	2
	Mismatch repair	1
	DNA replication	1
Z2X vs. Z2s	Antigen processing and presentation	3
	RNA transport	2
	Ribosome	2
Z2X vs. Z8X	Antigen processing and presentation	2
	RIG-I-like receptor signaling pathway	1
	Biotin metabolism	1
Z2X vs. Z12X	Antigen processing and presentation	3
	Peroxisome	1
Z6X vs. Z6s	Antigen processing and presentation	2
	RNA degradation	2
	RIG-I-like receptor signaling pathway	1
Z8X vs. Z8s	RIG-I-like receptor signaling pathway	1
	Methane metabolism	1
	Glycerolipid metabolism	1
Z10s vs. Z6s	Antigen processing and presentation	2
	Plant hormone signal transduction	2
Z10X vs. Z6X	Antigen processing and presentation	6
	Protein processing in endoplasmic reticulum	3
	Estrogen signaling pathway	2
Z10X vs. Z10s	Homologous recombination	2
	Antigen processing and presentation	2
	Fanconi anemia pathway	2
Z12X vs. Z12s	RIG-I-like receptor signaling pathway	1
	Biotin metabolism	1
	Fatty acid biosynthesis	1

## Data Availability

Data is contained within the article and Supplementary Material.

## Supplementary Materials

The following supporting information can be downloaded at: https://www.mdpi.com/article/10.3390/genes17091024/s1, Figure S1. Experimental design of mulberry grafting and phloem tissue sampling; Figure S2. Predicted stem-loop secondary structures of representative novel mulberry miRNAs; Figure S3. GO and KEGG functional enrichment of target genes for differentially expressed miRNAs; Figure S4. Correlation between differential miRNA expression and target gene qRT-PCR levels; Table S1. qRT-PCR Primer Information; Table S2. miRNA sequence statistics; Table S3. all sample annotation; Table S4. ncRNA Classification Statistics of Clean Reads; Table S5. Conserved miRNA; Table S6. Complete Annotation of Conserved miRNAs; Table S7. novel miRNA; Table S8. Complete Prediction Data of Novel miRNAs; Table S9. All Differentially Expressed miRNAs; Table S10. Complete List of Predicted Target Genes for Differentially Expressed miRNAs; Table S11. Full GO Enrichment Statistics; Table S12. KEGG Pathway Enrichment with Cross-Species Annotation Correction; Table S13. Pearson Correlation Between miRNA Abundance and Target Gene qRT-PCR Expression.
