# Supplementary material for: Identification and Analysis of Graft-Responsive miRNAs in Mulberry Rootstock–Scion Interactions
Source: Genes (Basel). 2026 Aug 28;17(9):1024. doi: 10.3390/genes17091024 (PMC13606142; doi:10.3390/genes17091024)
Supplement: Supplementary file 1 [file genes-17-01024-s001.zip › Supplementary Figures.pdf]

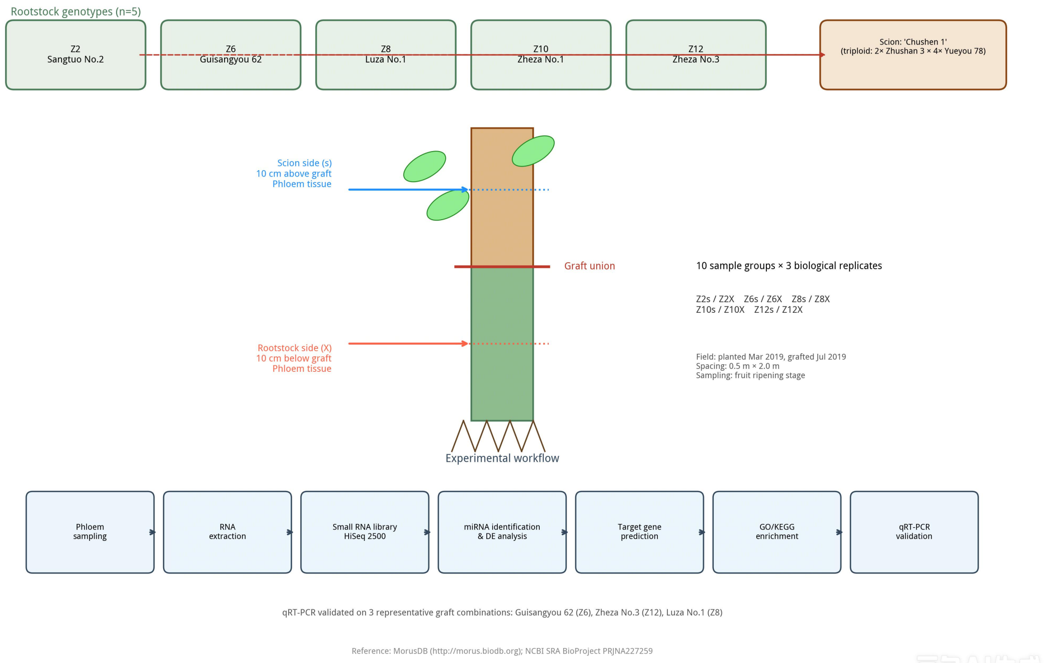


豆包AI生成

**Supplementary Figure S1. Experimental design of mulberry grafting and phloem tissue sampling**

*Figure Legend: Schematic workflow of mulberry grafting, phloem tissue sampling, and the experimental pipeline. Five mulberry rootstock genotypes (Sangtuo No.2, Guisangyou 62, Luza No.1, Zheza No.1, Zheza No.3) were grafted with the uniform triploid scion cultivar 'Chushen 1' (derived from 2× Zhushan 3 × 4× Yueyou 78). Phloem tissues were collected at 10 cm above (s, scion side) and 10 cm below (X, rootstock side) the graft union during the fruit ripening stage, generating 10 sample groups with three independent biological replicates each. Three representative graft combinations (Guisangyou 62, Zheza No.3, Luza No.1) were selected for subsequent qRT-PCR validation. This diagram illustrates genotype- and position-dependent miRNA expression differences and does not constitute direct evidence of long-distance inter-tissue miRNA transport across the graft junction. Experimental layout and mulberry genomic references follow standards established in MorusDB (http://morus.biodb.org) and NCBI SRA BioProject PRJNA227259.*

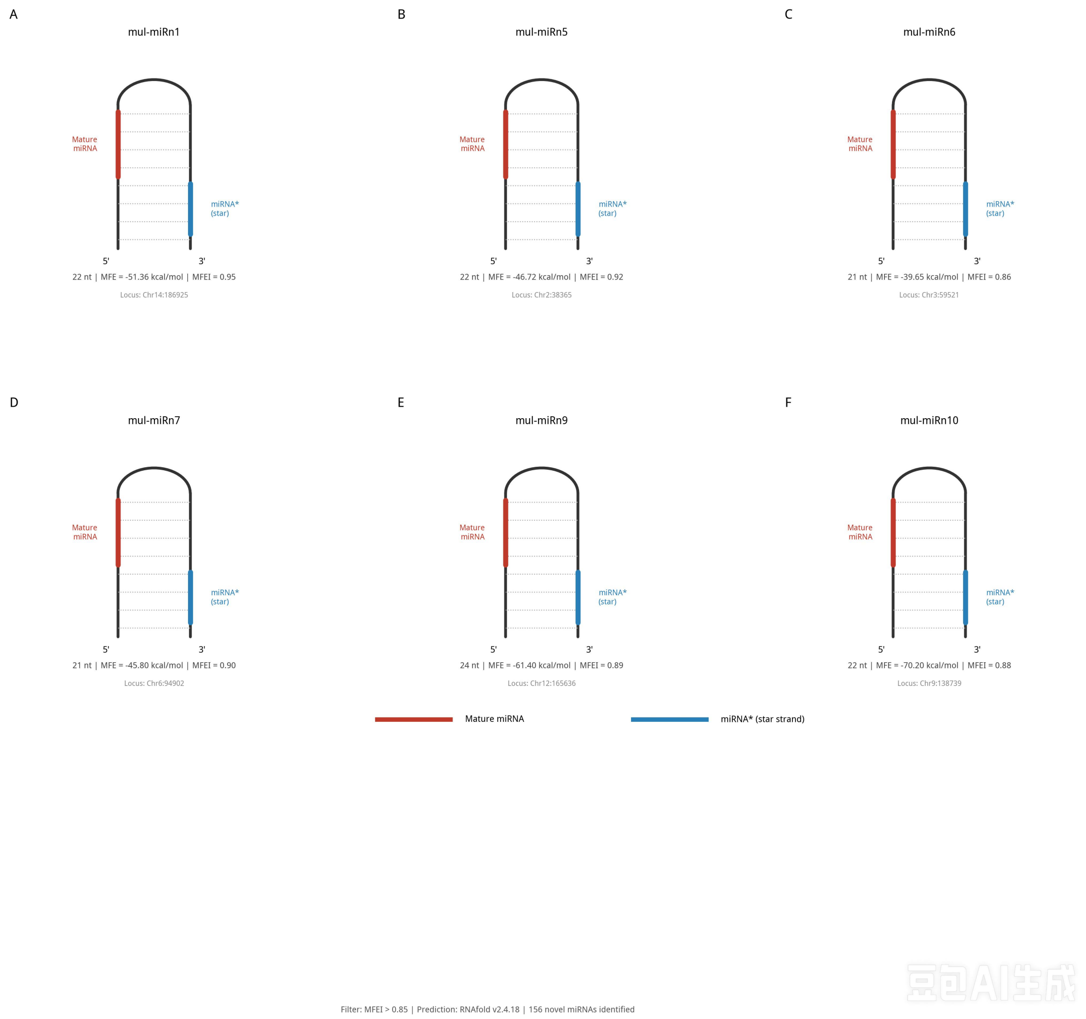


**Supplementary Figure S2. Predicted stem-loop secondary structures of representative novel mulberry miRNAs**

*Figure Legend: Predicted stem-loop secondary structures of six representative novel miRNAs identified in mulberry phloem tissues. Panels A–F correspond to mul-miRn1 (22 nt), mul-miRn5 (22 nt), mul-miRn6 (21 nt), mul-miRn7 (21 nt), mul-miRn9 (24 nt), and mul-miRn10 (22 nt), respectively. Mature miRNA sequences are highlighted in red; miRNA* (star) strands are highlighted in blue. Each structure is annotated with precursor length, minimum free energy (MFE, kcal/mol), MFE index (MFEI), and genomic locus. All novel miRNA precursors passed the inclusion threshold of MFEI > 0.85. Secondary structures were predicted using RNAfold v2.4.18. The 22 nt and 24 nt novel miRNAs may participate in secondary siRNA biogenesis or epigenetic regulation; further degradome sequencing or 5' RACE experiments are required to validate their direct cleavage activity. A total of 156 novel miRNAs were identified in this study, consistent with the 262 novel miRNAs previously reported in mulberry (Jia et al., 2014; BioProject PRJNA227259).*

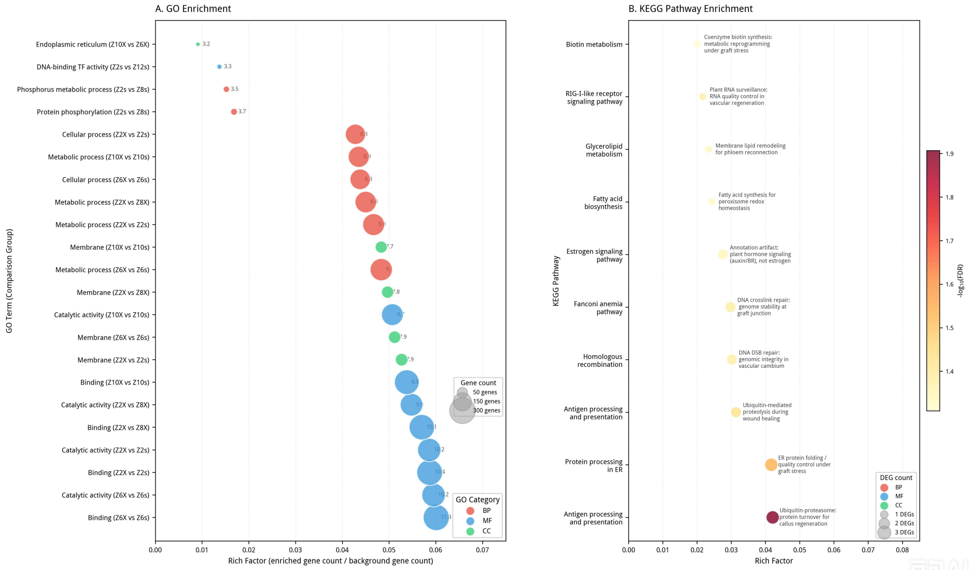


Note: Animal-derived KEGG pathway names (antigen processing, RIG-I, estrogen, Fanconi anemia) are cross-species orthology annotations. In mulberry, these correspond to ubiquitin-proteasome turnover, RNA surveillance, plant hormone signaling, and DNA repair pathways — not animal immune or disease functions. Background gene set: MorusDB mulberry whole-genome annotation (Morus notabilis).

**Supplementary Figure S3. GO and KEGG functional enrichment of target genes for differentially expressed miRNAs**

*Figure Legend: Bubble plots of Gene Ontology (GO) and KEGG pathway enrichment for target genes of differentially expressed miRNAs. (A) GO enrichment analysis separated into Biological Process (BP, red), Molecular Function (MF, blue), and Cellular Component (CC, green). Bubble size corresponds to the number of enriched target genes; numbers adjacent to bubbles indicate −log10(FDR) significance. The most significantly enriched terms include binding, catalytic activity, metabolic process, cellular process, and membrane component, consistent with active transcriptional and metabolic reprogramming during graft union formation. (B) KEGG pathway enrichment across all comparison groups. Bubble color represents −log10(FDR); bubble size represents the number of differentially expressed genes (DEGs). Animal-derived pathway names — including antigen processing and presentation, RIG-I-like receptor signaling, estrogen signaling, and Fanconi anemia pathway — are cross-species orthology annotations only. In mulberry, these pathways correspond to ubiquitin–proteasome protein turnover, intracellular RNA surveillance, plant hormone (auxin/brassinosteroid) signaling, and DNA damage repair during graft wound healing and vascular reconnection, rather than animal immune or disease functions. All enrichment analyses used the MorusDB mulberry whole-genome annotation (Morus notabilis) as the background gene set.*

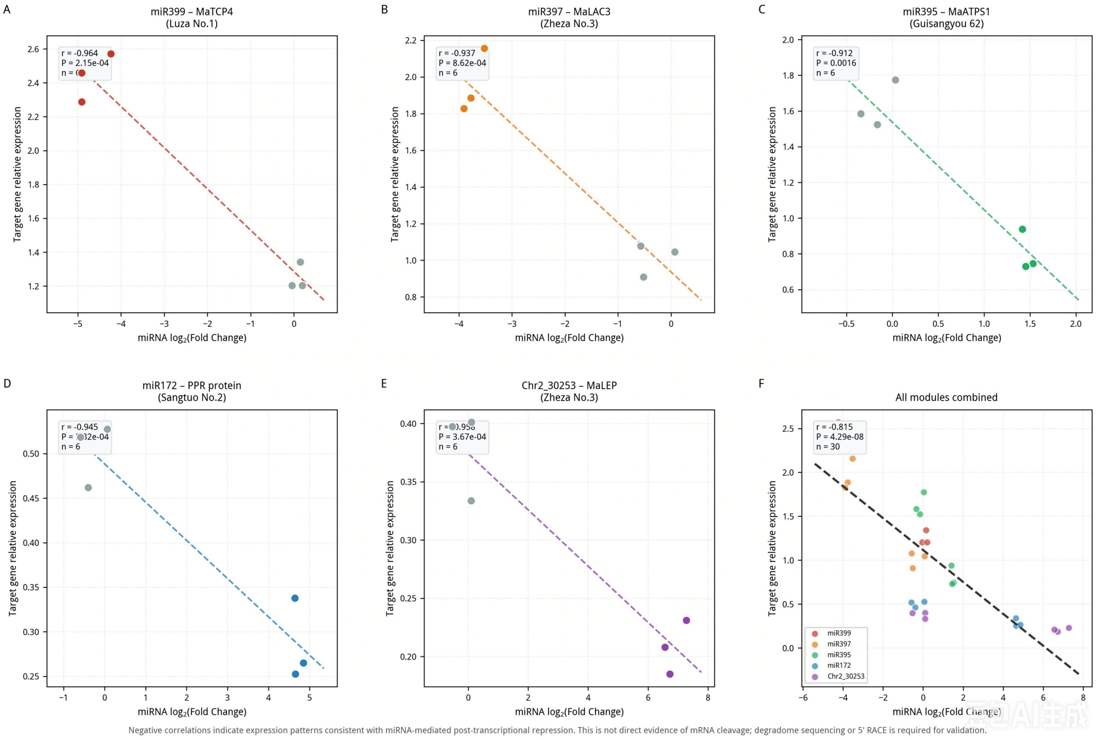


**Supplementary Figure S4. Correlation between differential miRNA expression and target gene qRT-PCR levels**

*Figure Legend: Correlation scatter plots between miRNA log2 fold change and qRT-PCR-quantified target gene relative expression. Panels A–E show five core miRNA–target regulatory modules separately: (A) miR399–MaTCP4 (Luza No.1, $r = -0.964$, $P = 2.15 \times 10^{-4}$); (B) miR397–MaLAC3 (Zheza No.3, $r = -0.937$, $P = 8.62 \times 10^{-4}$); (C) miR395–MaATPS1 (Guisangyou 62, $r = -0.912$, $P = 1.63 \times 10^{-3}$); (D) miR172–PPR protein (Sangtuo No.2, $r = -0.945$, $P = 5.32 \times 10^{-4}$); (E) Chr2_30253–MaLEP (Zheza No.3, $r = -0.958$, $P = 3.67 \times 10^{-4}$). Panel F shows the combined correlation across all modules ($r = -0.815$, $P = 4.29 \times 10^{-8}$, $n = 30$). Gray dots represent control samples; colored dots represent treatment samples. Dashed lines indicate linear regression fits. All modules exhibited significant negative correlations, consistent with the predicted miRNA-mediated post-transcriptional repression model. However, these expression correlations do not constitute direct molecular evidence of miRNA-guided mRNA cleavage; degradome sequencing or 5′ RLM-RACE validation is required. Notably, MaATPS1 (Morus013341) has been independently validated as a target of miR-395a by 5′ RLM-RACE (Wu et al., 2015).*

## References Cited in Figure Legends


*1.He, N., Zhang, C., Qi, X., et al. (2013). Draft genome sequence of the mulberry tree Morus notabilis. Nature Communications, 4, 2445.*

*2.Jia, Q., Xiao, L., Sun, W., et al. (2014). Small RNA profiling in two mulberry varieties identifies miRNAs involved in salt and drought stress. Plant Cell Reports, 33(12), 1963–1976.*

*3.Li, T., Li, M., Wei, J., et al. (2014). MorusDB: a resource for mulberry genomics and genome biology. Database, 2014, bau054.*

*4.Wu, B., Wang, J., Li, M., et al. (2015). Identification and characterization of microRNAs and their targets in mulberry (Morus multicaulis) by high-throughput sequencing and degradome analysis. Plant Cell Reports, 34(12), 2141–2156.*

*5.Gai, Y., Guo, X., Ji, M., et al. (2018). Identification of miRNAs and their targets in mulberry under drought stress using high-throughput sequencing and degradome analysis. Plant Physiology and Biochemistry, 127, 183–194.*
